# Co-Delivery of mRNA and pDNA Using Thermally Stabilized Coacervate-Based Core-Shell Nanosystems

**DOI:** 10.3390/pharmaceutics13111924

**Published:** 2021-11-13

**Authors:** Sarah S. Nasr, Sangeun Lee, Durairaj Thiyagarajan, Annette Boese, Brigitta Loretz, Claus-Michael Lehr

**Affiliations:** 1 Helmholtz Institute for Pharmaceutical Research Saarland (HIPS), Helmholtz Centre for Infection Research (HZI), Saarland University, 66123 Saarbrücken, Germany; Sara.Nasr@helmholtz-hips.de (S.S.N.); Sangeun.Lee@helmholtz-hips.de (S.L.); Thiyagarajan.Durairaj@helmholtz-hips.de (D.T.); Annette.Boese@helmholtz-hips.de (A.B.); Claus-Michael.Lehr@helmholtz-hips.de (C.-M.L.); 2 Department of Pharmacy, Saarland University, 66123 Saarbrücken, Germany; 3 Department of Pharmaceutics, Faculty of Pharmacy, Alexandria University, Alexandria 21521, Egypt

**Keywords:** nucleic acid vaccine, complex coacervation, nanocarriers, anisotropic nanogel, physical cross-linking, dual loading

## Abstract

Co-delivery of different species of protein-encoding polynucleotides, e.g., messenger RNA (mRNA) and plasmid DNA (pDNA), using the same nanocarrier is an interesting topic that remains scarcely researched in the field of nucleic acid delivery. The current study hence aims to explore the possibility of the simultaneous delivery of mRNA (mCherry) and pDNA (pAmCyan) using a single nanocarrier. The latter is based on gelatin type A, a biocompatible, and biodegradable biopolymer of broad pharmaceutical application. A core-shell nanostructure is designed with a thermally stabilized gelatin–pDNA coacervate in its center. Thermal stabilization enhances the core’s colloidal stability and pDNA shielding effect against nucleases as confirmed by nanoparticle tracking analysis and gel electrophoresis, respectively. The stabilized, pDNA-loaded core is coated with the cationic peptide protamine sulfate to enable additional surface-loading with mRNA. The dual-loaded core-shell system transfects murine dendritic cell line DC2.4 with both fluorescent reporter mRNA and pDNA simultaneously, showing a transfection efficiency of 61.4 ± 21.6% for mRNA and 37.6 ± 19.45% for pDNA, 48 h post-treatment, whereas established commercial, experimental, and clinical transfection reagents fail. Hence, the unique co-transfectional capacity and the negligible cytotoxicity of the reported system may hold prospects for vaccination among other downstream applications.

## 1. Introduction

Nucleic acid-based therapies are currently moving with vast strides towards increasingly broader clinical application. DNA and various forms of RNA (siRNA, miRNA, mRNA, and saRNA), as well as antisense oligonucleotides [1,2], have shown promise in alleviating various genetic disorders previously uncatered for by conventional therapeutics. Investigating nucleic acids (NA) as vaccination tools has been for years one of the most advanced fields of research for nucleic acid-based therapies. Many ongoing clinical trials investigate mRNA-based vaccines for rabies, influenza H7N9, influenza H10N8, cytomegalovirus, human metapneumovirus, parainfluenza virus 3, respiratory syncytial virus, and Zika, among others [3]. Moreover, several veterinary DNA vaccines have already been approved [4]. Nevertheless, until recently, no NA-based vaccines have been approved for human use. This situation has been rapidly changing following the outbreak of SARS-CoV-2 in 2019. In December 2020, the mRNA vaccines of Pfizer/BioNTech (BNT162b2) [5] and Moderna (mRNA-1273) [6] were the first vaccines to receive approval for emergency use in humans against SARS-CoV-2.

Given the physicochemical properties of mRNA and its inherent instability that renders its intact, intracellular translocation, and successful translation quite challenging, the life-saving impact of such mRNA-based vaccines was mostly possible via their incorporation into efficient nanocarriers [7,8]. In the past decades, several nanocarriers have been developed for the delivery of either mRNA, siRNA, or pDNA. Often, the carrier would show success in discrete delivery of more than one NA species with minor modifications from one species to the other [9,10,11,12]. Yet very little research has so far been dedicated to the simultaneous delivery of more than one NA species on the same carrier [13,14,15]. In 2018, Ball et al. demonstrated the possibility of co-delivering mRNA and siRNA on one lipidoid nanoparticle (LNP) carrier, highlighting the possibility and value of combining gene silencing with protein replacement therapeutic approaches [12,13]. In the present study, we aim to investigate the scarcely explored possibility of the simultaneous delivery of two classes of nucleic acid, namely mRNA and pDNA, on the same carrier, to profit from the complementary advantages of both NAs.

Messenger RNA, while being structurally less stable than pDNA, if it can successfully evade endo-lysosomal digestion and reach the cytosol in an intact form, it can be rapidly translated in the cytosol without the need for nuclear translocation [13]. As opposed to pDNA, mRNA offers rapid onset, transient cytosolic protein and peptide expression, as well as a higher transfection efficiency that is cell cycle independent. Another feature of mRNA as a vaccination tool is its self-adjuvanting property. RNA has been repeatedly reported to curb its own translatability via type I interferon or toll-like receptor (TLR) inducible mechanisms [16,17,18,19,20], thus promoting innate at the expense of adaptive immunity [21,22]. Hence, the use of modified hypoimmunogenic mRNA (e.g., O-methylated cap analogs and chemically modified bases such as pseudouridine) [17], and 5-methyl-cytidine limits the self-adjuvanting property of mRNA enhancing and promotes its translation. Yet, this poses the need for the co-delivery of adjuvants that can discretely improve the immune response to mRNA-encoded antigens while avoiding deleterious effects to mRNA’s translatability [20,23]. The co-delivery of such adjuvants in NA format may be convenient in such a case, to ensure the co-delivery of both adjuvant and antigen to the same target cell(s). Nanocarrier systems can enhance intracellular delivery, which will allow in-vivo antigen-presenting cells targeting [24]. Co-delivery of antigen and adjuvant can help in vaccine dose sparing and prevent loss of time synchronization between adjuvant and the vaccine, where adjuvant delivery to non-primed antigen-presenting cells may lead to an autoimmune reaction [25].

Examples of the importance of such targeted co-delivery can be found in the context of anti-cancer vaccines, where studies have demonstrated that untargeted delivery of NA-based adjuvants, such as TLR4 and CD40 encoding mRNA, caused an enhancement in tumor resistance to tumor-specific cytotoxic T cells and neoangiogenesis, respectively [26,27]; while the precise ex-vivo electroporation of these exact adjuvants into immunosuppressed tumor-infiltrating DCs restored their antigen-presenting potential to promising results [28,29]. In this case, targeted expression of the adjuvant by DCs becomes instrumental and, so far, cannot be achieved by mRNA sequence manipulation.

Plasmid DNA, on the other hand, while being more challenging to deliver and translate due to the additional requirement of crossing the nuclear membrane [30], has a longer half-life than mRNA and presents more chances for manipulation and control over its rate, duration, and expressing cell type [31,32]. pDNA’s promoter manipulation can be used to alter the kinetics of pDNA expression [33]; such tuning can prove much harder to achieve using mRNA as it will require careful optimization of the tRNA frequency of each codon on the open reading frame [34]. Promotor manipulation can also be used to exclude pDNA expression to certain cell types [31,32,35]. Several studies have demonstrated the possibilities of transcriptionally targeting dendritic cells (DCs) using a range of promotors associated with DC-specific genes encoding for Fascin [31], DC-SIGN, DC-STAMP, and Langerin [32].

Co-delivering mRNA and pDNA can benefit from the rapid onset, transient expression of mRNA, and the adjustable expression of pDNA, creating distinct expression patterns for one or more transgenes. Such an approach could be of value in achieving sequential expression of the same therapeutic protein at two different time points analogous to conventional multi-dosing or as a tool for co-expression of synergistic therapeutic proteins and/or peptides with varying half-lives or expression target cells. Within a vaccination context, this could mean a prolonged antigen expression or co-expression of body-own immunostimulatory protein(s) as an adjuvant to enhance vaccine efficacy.

For simultaneous co-delivery of mRNA and pDNA using just one and the same nanocarrier, we first prepared gelatin–pDNA coacervates. It is important to set this work apart from that of Morán et al., who reported a system based on gelatin type B instead of gelatin type A, and relied on electrostatic interaction between the negatively charged gelatin B and protamine to encapsulate the nucleic acid species. This system could be formulated either in the absence or presence of the NA cargo [36]. The NA was in their case a non-functional model cargo (Torula yeast RNA). On the other hand, in the current study gelatin type A was selected given its positive charge and therefore its capacity for spontaneous assembly with DNA, as well as its lack of antigenicity being a denaturation product of collagen and hence its suitability for multi-dosing [37]. pDNA on the other hand plays a double role: (1) a combination of pDNA with gelatin type A in the optimum ratio leads to the formation of a gelatin–pDNA coacervate (CoAc) core possessing unique thermal properties, and (2) pDNA also serves as a biologically active NA cargo. In the second step, we implemented thermal rather than chemical stabilization of the CoAc to form a stable gel-based particle (TS-CoAc), which enables the subsequent coating of the gelatin–pDNA core with protamine sulfate. This structure benefits from protamine’s nuclear translocation amino acid sequences, thus maximizing pDNA’s transfection efficiency [38,39]. At the same time, the resulting positive surface charge allows for surface-loading of mRNA and facilitates particle-cell interaction [40]. As proof of principle for co-transfection, fluorescent reporter proteins encoded by mRNA (mCherry) and pDNA (pAmCyan) were used.

## 2. Materials and Methods

### 2.1. Materials for Nanocarriers and Controls

Gelatin GELITA^®^ MedellaPro^®^ < 100, porcine gelatin, 228 g Bloom, pharmaceutical-grade was purchased from GELITA^®^ Deutschland GmbH, Eberbach, Germany. Protamine sulfate was purchased from Sigma-Aldrich, Darmstadt, Germany. JetMessenger (JetM) and JetPrime (JetP) were purchased from Polyplus-transfection^®^, Illkirch, France. Branched polyethyleneimine (PEI), M_w_~25,000, was purchased from Sigma-Aldrich Darmstadt, Germany. Lipofectin was purchased from Invitrogen, Thermo Fisher Scientific, Darmstadt, Germany. Purified water was obtained from a Milli-Q water purification system (Merck Millipore, Darmstadt, Germany), and is referred to as MQ water. DLin-MC3-DMA was purchased from MedChemExpress (Middlesex County, NJ, USA), Cholesterol was purchased from Sigma-Aldrich Darmstadt, Germany, DSPE-PEG 2000 and DPPC were a kind gift from Lipoid GmbH, Ludwigshafen, Germany.

### 2.2. Materials for Analytics

Agarose research grade was purchased from Serva^®^, Heidelberg, Germany. Disodium dihydrate ethylenediamine tetra-acetic acid (EDTA-Na_2_) was purchased from Roth GmbH+Co. KG, Karlsruhe, Germany. DNA loading dye (6×) was purchased from Thermo Fisher Scientific, Waltham, MA, USA. DNA Ladder 250–10,000 bp was purchased from PEQLAB Biotech GmbH, Erlangen, Germany. Ethidium bromide 10 mg·mL^−1^ was purchased from Sigma-Aldrich, Darmstadt, Germany. Live/dead fixable stain (568/583) was purchased from PromoCell GmbH, Heidelberg, Germany. Quant-iT™ PicoGreen™ dsDNA Assay Kit and RiboGreen™ RNA Assay-Kit, DNase I, DNase I buffer, 50 mM EDTA, RNase A, and Ribolock were purchased from Thermo Fisher Scientific, Darmstadt, Germany. Bovine collagen type I solution, Purecol was purchased from CellSystems, Troisdorf, Germany.

### 2.3. Nucleic Acids

Plasmid DNA encoding AmCyan fluorescent protein (pAmCyan1-C1) was purchased from Clontech Laboratories Inc., Mountain View, CA, USA. The plasmid was propagated in Subcloning Efficiency™ DH5α E. coli competent cells (Invitrogen, Thermo Fisher Scientific, Darmstadt, Germany), then isolated and purified using Qiagen EndoFree Plasmid Mega Kit (Qiagen, Hildesheim, Germany) as per manufacturer’s protocol. CleanCap^®^ mCherry mRNA was purchased from Tri-Link BioTechnologies LLC, San Diego, CA, USA.

### 2.4. Cell Culture

Murine dendritic cell line DC2.4 was purchased from Millipore Corporation, California, USA. Cells were cultured in RMPI-1640 supplemented with 10% FCS, 1× non-essential amino acids (NEAA, 100×), 1× HEPES buffer solution (1M), and 0.0054× β-mercaptoethanol 100×, purchased from Merck, Darmstadt, Germany. RPMI-1640, FCS, NEAA, Trypsin-EDTA (0.25%), and HEPES were all purchased from Gibco, Thermo Fisher Scientific, Darmstadt, Germany.

### 2.5. Particle Core Assembly

**Figure 1:**
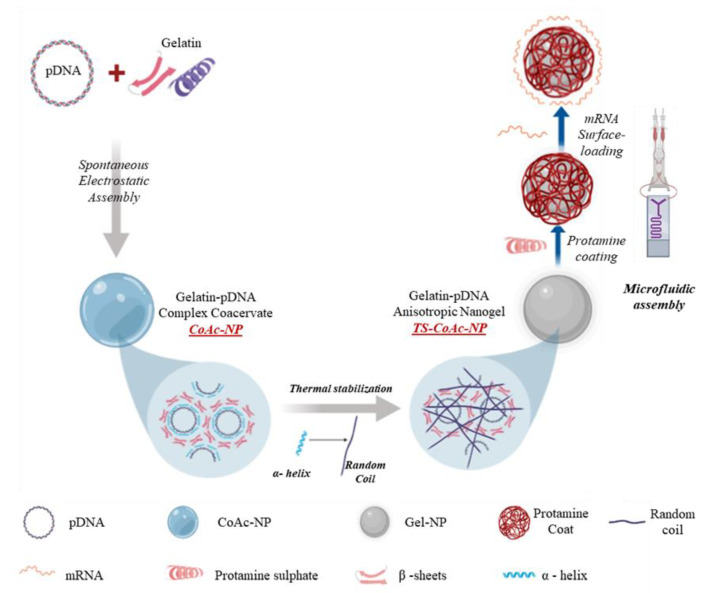
The general preparation procedure of CoAc, TS-CoAc, and P-TS-CoAc, and the proposed mechanism of thermal stabilization of CoAc into TS-CoAc. P-CoAc was prepared by the introduction of CoAc and protamine sulfate into a microfluidic coating system while skipping the thermal stabilization step of CoAc.

Gelatin–pDNA coacervates (CoAc) were prepared using the complex coacervation technique (Figure 1), where all coacervates were assembled in MQ water. First, the optimum mixing temperature of gelatin and pDNA was assessed by mixing gelatin type A solution (10 mg·mL^−1^) with pAmCyan (100 µg·mL^−1^) in a ratio of 1:1 *v*/*v* under three different mixing temperatures (23, 37 and 55 °C). For further optimization of the mixing conditions, the appropriate mass ratio between gelatin and pDNA was investigated. A solution of pAmCyan in MQ water (100 µg·mL^−1^) was added to gelatin type A solutions in MQ water with different concentrations (10 mg·mL^−1^, 7 mg·mL^−1^, 5 mg·mL^−1^, 3 mg·mL^−1^, 1 mg·mL^−1^ and 0.1 mg·mL^−1^) at 37 °C (Table 1). The two solutions were then mixed via vortexing at maximum speed for 1 min. Gelatin–pDNA coacervates (CoAc) were thus assembled at gelatin to pDNA mass ratios of 100:1, 70:1, 50:1, 30:1, 20:1 and 1:1, respectively (Table 1). Assessments of the optimum mixing ratio and temperature were performed using dynamic light scattering measurements of particle size and zeta potential.

### 2.6. Thermal Particle Core Stabilization

CoAc_30_ was selected given its small size, low PDI, and negative zeta potential for further experiments. Thermal stabilization was performed in four subsequent heating–cooling cycles per sample. Each cycle started with CoAc incubation at 55 ± 0.5 °C for 30 min, followed by rapid cooling to 0 ± 0.5 °C for 5 min. The resulting gelated particle is referred to as thermally stabilized coacervate (TS-CoAc), while particles that were not subjected to thermal cycling (unstabilized) are referred to simply as coacervate (CoAc).

### 2.7. Shell Deposition and mRNA Loading

Protamine sulfate solution (0.3 mg·mL^−1^) was used to coat the preformed CoAc_30_ or TS-CoAc_30_, to the final protamine sulfate to gelatin mass ratio of 1:5 *w*/*w* (taking into consideration the two-fold dilution of gelatin’s mass contribution during the coacervation step). Protamine sulfate solution was mixed with the preformed particle cores under laminar flow conditions in a microfluidic setting, using a meander chip with two inlets, one for each component at a total flow rate of (2 mL·min^−1^). The formed core-shell system was left to stand for at least 24 h at 4 °C. The core-shell particle was then surface-loaded with 1 µg mCherry per 170 µg of particles, and mixed by simple pipetting to a final pDNA: mRNA mass ratio of 5:1 per particle. The particles were further allowed to stand for 15 more minutes before use.

### 2.8. Dynamic Light Scattering (DLS)

Samples were characterized for particle size, PDI, and zeta potential using Zetasizer Nano-ZS (Malvern Instruments, Worcestershire, U.K.), utilizing 4 mW He−Ne laser at a wavelength of 633 nm and a backscattering angle of 173° at 25 °C. Samples were measured in concentrations of (1550 µg·mL^−1^) of non-coated particles and (925 µg·mL^−1^) of coated particles. Particle size is given as intensity-based z-average. Moreover, the colloidal stability of P- CoAc and P- TS-CoAcs was studied for three weeks at 4 °C storage temperature, where dynamic light scattering was used to assess changes in particle size or PDI over time.

### 2.9. Nanoparticle Tracking Analysis (NTA)

NTA (NanoSight LM10, Malvern Instruments, Worcestershire, UK) was used to assess the effect of thermal stabilization on the colloidal stability of the gelatin–pDNA core in a cell culture medium; this is where both non-stabilized CoAc and thermally stabilized TS-CoAc were incubated in RPMI-1640, in a ratio of (1:10 *v*/*v*) at 37 °C for 4 h. For each sample, particle count (particle·mL^−1^) was recorded at zero time and after 4 h of incubation in RPMI-1640. Data was collected from three videos, 30 s each, where the camera level was manually set to 14 during all captures. NanoSight 3.3 software was used to process the videos at a detection threshold of 5.

The number of nanoparticles·mL^−1^ was also determined in MQ water using the same settings. The information was used along with the previous knowledge of the number of either pDNA or mRNA molecules per 1 mL of particle suspension to determine the numbers of pDNA and mRNA molecules per nanocarrier, based on the method previously described by Zagato et al. [41].

### 2.10. Circular Dichroism (CD)

Gelatin solution (3 mg·mL^−1^) was prepared by dissolving gelatin in MQ water at 55 °C. From the gelatin stock, CoAc was prepared as previously described. A portion of the resultant CoAc was then thermally cycled to prepare TS-CoAc, as previously described. CD spectra of all samples were recorded at 37 °C on a Jasco 810 spectropolarimeter (Jasco, Tokyo, Japan) in a 0.1-cm path-length quartz cell. Samples were scanned in and blanked to MQ water. Each sample was scanned 15 rounds per measurement, and eventually, the spectra for each were obtained after subtracting the contribution of the MQ water blank.

### 2.11. Transmission Electron Microscopy (TEM)

Unstained CoAc and TS-CoAc particles, with or without protamine sulfate coating, were visualized using TEM (JEM 2011, JEOL, St Andrews, UK). Either non-coated particles (775 µg·mL^−1^) or coated particles (925 µg·mL^−1^) were used, where 10 µL samples were mounted on a copper grid (S160-4, Plano GmbH, Wetzlar, Germany), and allowed to dry overnight before visualization. The TEM measurement was performed at an accelerating voltage of 200 kV.

### 2.12. Agarose Gel Electrophoresis

Investigating the effect of thermal stabilization on pDNA protection was performed by Dnase I challenge, comparing P-CoAc without and with thermal stabilization. In the same assay, the protection of the surface-bound mRNA against RnaseA was also monitored. mRNA-loaded P-TS-CoAc or P-CoAc (NA load of 1.5 µg mRNA and 7.5 µg pDNA) were incubated with 0.008 U·mL^−1^ Dnase I and 0.027 µg·mL^−1^ Rnase A (both Sigma-Aldrich, Darmstadt, Germany) in Dnase I working buffer at 37 °C for 30 and 60 min. Particle samples were compared to samples of equivalent masses of naked mRNA and pDNA subjected to the same treatments. Following the pre-stated incubation periods, 20 µL samples or controls were drawn from the reaction mixture and the reaction was quenched using 3 µL 50 mM EDTA and 1 µL Ribolock (both Sigma-Aldrich, Darmstadt, Germany), then the nucleic acids were released from the particles via digestion with Trypsin (30 µL, 1.17 mg·mL^−1^) for 30 min at 37 °C, followed by addition of high molecular weight Heparin (10 µL, 300 mg·mL^−1^). Samples were loaded onto 1.3% *w*/*v* agarose gel (Serva, Heidelberg, Germany) containing ethidium bromide (0.3 µg·mL^−1^) (Sigma-Aldrich, Darmstadt, Germany), in TBE buffer (1×) and run for 90 min at 90 mV. The gel was visualized under UV-light (Fusion FX7 imaging system, Peqlab, Erlangen, Germany).

As further confirmation of the difference in pDNA shielding effects between thermally stabilized and unstabilized cores in a more physiologically relevant medium, sample volumes from uncoated CoAc and TS-CoAc equivalent to 3 µg pDNA were incubated with 10% FCS in HBSS for 3 h at 37 °C. The activity of serum nucleases was then quenched using EDTA (150 µL, 50 mM). pDNA was then released from the sample using subsequent treatment with Trypsin (30 µL, 1.17 mg·mL^−1^) for 150 min at 37 °C, then in a high molecular weight Heparin solution (30 µL, 30 mg·mL^−1^). Samples were loaded onto a 0.7% *w*/*v* agarose gel and run for 60 min at 60 mV. Data were normalized to intact supercoiled pDNA as a control.

### 2.13. PicoGreen and RiboGreen Assays

To assess the entrapment efficiency of pDNA and mRNA, CoAc_30_ or TS-CoAc_30_ were ultracentrifuged at 58,000× *g*, 4 °C for 2 h. The supernatant was then analyzed for pAmCyan1 content using PicoGreen assay or for mCherry using RiboGreen assay, according to the manufacturer’s protocols. Data were normalized to free pAmCyan or mCherry subjected to the same treatment as the samples.

### 2.14. In-Vitro Biological Assessment of the Nanocarrier

Both transfection efficiency and cytotoxicity of the system were assessed in murine dendritic cell line DC2.4. Transfection efficiency of pAmCyan and mCherry was assessed in DC2.4 murine dendritic cell line, passages 6 to 8. Briefly, cells were seeded at a density of 50,000 cells per well in 24 well plates, in RPMI-1640 containing FCS (10% *v*/*v*), HEPES (1%), NEAA (1%), β-mercaptoethanol (0.0054%). Cells were grown for 48 h reaching confluency of approximately 80% before being treated with either samples or controls. Samples were either P-CoAc or P-TS-CoAc, both using a concentration of 170 µg particles per well, equivalent to NA concentrations of 1 µg mCherry and 5 µg pAmCyan per well. As negative controls, untreated cells and cells treated with either naked mRNA or pDNA were used. Cells treated with commercial transfection reagents were used as positive controls. For single transfection, JetMessenger was used for mRNA, whereas JetPrime was used for pDNA. As an additional positive control, a solid lipid nanoparticle (SLN) inspired by the current clinical standard of mRNA/siRNA delivery was prepared. Briefly, an aqueous solution of mRNA and pDNA in a mass ratio of 1:5 (pH = 4) was mixed with an ethanolic solution of the following lipids, DLin-MC3-DMA, DPPC, Cholesterol, and DSPE-PEG2000, in a molar ratio of 50:10:38.5:1.5 and at a final N/P ratio of 0.5 [42]. Both JetPrime and JetMessenger along with Lipofectin, polyethyleneimine, and solid lipid nanoparticles were used as controls using a combination of both pDNA and mRNA to assess their dual-transfection efficiency. JetPrime, JetMenssenger, and Lipofectin are commercial transfection reagents. JetMessenger is optimized for mRNA, and JetPrime is recommended for pDNA. Lipofectin can be used for both species. These commercial reagents were used per the manufacturer’s protocols. High M_W_ branched PEI is a golden standard among the polymers in terms of transfection efficacy, despite its cytotoxicity. Thus, PEI-NPs were prepared and included as a polymeric reference sample, using PEI: pDNA: mRNA mass ratio equivalent to the protamine sulfate: pDNA: mRNA mass ratio of 30:5:1 originally present in TS-CoAc. As an internal control, the same protamine sulfate concentration that was used for CoAc or TS-CoAc coating was used to formulate a protamine sulfate coacervate with pDNA, to which mRNA was added immediately before cell treatment, in the same pDNA: mRNA mass ratio used for either TS-CoAc or CoAc. Samples were incubated with cells for 6 h under shaking at 250 RPM, then removed and replaced with fresh medium and further incubated for 48 h. Cell harvesting was performed following washing twice with HBSS, where cells were detached using Trypsin-EDTA (100 µL), followed by the addition of 2% FCS in HBSS (900 µL). Samples were centrifuged at 4 °C and 300× *g* for 5 min, the pellet was rewashed in 1 mL HBSS then re-suspended and fixed in paraformaldehyde (4% *w*/*v*). Transfection efficiency was analyzed using flow cytometry (BD LSRFortessa^TM^ Cell Analyzer Biosciences, Heidelberg, Germany), using the PE-Texas red channel for mCherry and AmCyan channel for pAmCyan. Flowjo version 10.6.1 was used for data processing.

Cytotoxicity of P-TS-CoAc was assessed in DC2.4, passages 10 to 12. Cells were seeded as previously described and then treated with P-TS-CoAc in a concentration of (340 µg·mL^−1^, 170 µg·mL^−1^, or 85 µg·mL^−1^). Cells were incubated with the samples for 6 h under shaking at 250 RPM, following which the samples were removed, and the cells detached as previously described. As a positive control, cells killed by heating at 70 °C post detachment were used. Following the last washing step and before fixation with paraformaldehyde (4% *w*/*v*), cells were stained with live dead fixable stain 568/583 (PromoCell GmbH, Heidelberg, Germany) according to the manufacturer’s protocol. The used kit stains only dead cells using cell membrane-impermeable amine-reactive peptides that can be detected on the PE-emission filter. The percentage of dead cells in the different samples was then measured by flow cytometry using the PE-A channel. Cell viability was calculated according to Equation (1).

(1) $$\mathrm{Cell}\;\mathrm{viability}\;\left(\%\right)\;=\;\left(\frac{\mathrm{total}\;\mathrm{cell}\;\mathrm{number}\;-\;\mathrm{PE}\;\mathrm{positive}\;\mathrm{cell}\;\mathrm{number}}{\mathrm{total}\;\mathrm{cell}\;\mathrm{number}}\right)\times 100$$

### 2.15. Confocal Laser Scanning Microscopy (CLSM)

DC2.4 cells were seeded in 8 well glass bottom µ-slide (Ibidi, Gräfelfing, Germany), precoated with 1 mg/mL bovine collagen type I solution, Purecol (CellSystems, Troisdorf, Germany), at a density of 25,000 cells/well. The transfection procedure was performed as previously described. Immediately before visualization, the cells were washed twice using HBSS and fixed for 5 min with 4% (*v*/*v*) paraformaldehyde (PFA; Electron Microscopy Sciences) in HBSS for 5 min at r.t. After rinsing with HBSS, cells were mounted and stored at 4 °C until CLSM analysis (Leica TCS SP8, Leica Microsystems, Mannheim, Germany). Image acquisition was conducted on a Leica TCS SP8 confocal imaging microscope with a 25× water immersion objective (Fluotar VISIR 25×/0.95) at 1024 × 1024 resolution. For AmCyan, fluorescence was detected between 495–550 nm (excited at 405 nm; 24% laser intensity), for mCherry, fluorescence was detected between 683–784 nm (excited at 561 nm; 10% laser intensity), both using a HyD detector. Images were then processed with the Leica Application Suite (LAS) X software.

### 2.16. Statistical Analysis

Data were analyzed using Graph Pad Prism 8 for Windows (Version 8.01, GraphPad Software Inc., San Diego, CA, USA) and generally presented as the mean of individual values (generally 3–9 samples), with standard deviation indicated by the error bars. (N) refers to the number of experiments, (n) refers to the number of samples per experiment. A one-way ANOVA was performed for all test samples, followed by Tukey’s post hoc test for assessment of inter-group individual differences. Data were considered statistically significant at a level of significance of *p* < 0.05 (* *p* < 0.05, ** *p* < 0.01, *** *p* < 0.001 and **** *p* < 0.0001).

## 3. Results and Discussion

### 3.1. Particle Preparation

Either non-stabilized coacervates (CoAc) or thermally stabilized coacervates (TS-CoAc) were prepared according to the scheme illustrated in (Figure 1), with the sample compositions indicated in (Table 1). Thermal stabilization was performed as four subsequent heating–cooling cycles (55 ± 0.5 °C for 30 min followed by 0 ± 0.5 °C for 5 min per cycle). Subsequent protamine sulfate coating was performed via a microfluidic assembly to give protamine sulfate coated TS-CoAc (P-TS-CoAc).

#### 3.1.1. Core Assembly

**Figure 2:**
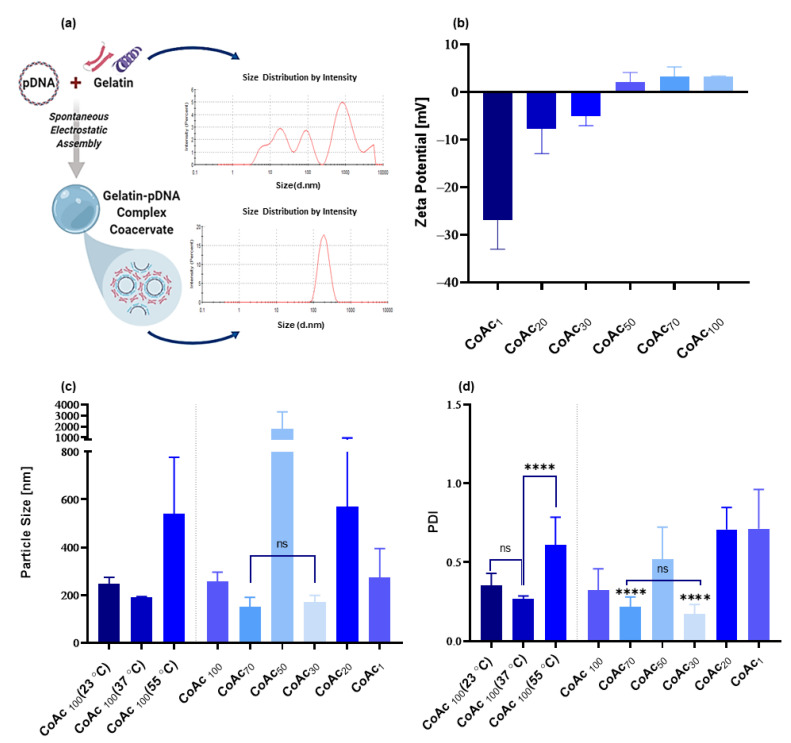
Assessment of the impact of gelatin: pAmCyan mass ratios (of 100, 70, 50, 30, 20, and 1) and coacervate assembly temperature (at 23, 37, and 55 °C) on resulting CoAc using dynamic light scattering. (**a**) The particle size distribution of gelatin A before and after coacervation with pAmCyan in MQ water. (**b**) Zeta potential (mV) of CoAcs (*N* = 2, *n* = 2). (**c**) Particle size (nm) of CoAcs assembled at 37 °C and of CoAc_100_ assembled at 23, 37 and 55 °C (*N* = 3, *n* = 3) (**d**) Polydispersity index (PDI) of CoAcs assembled at 37 °C and PDI of CoAc_100_ assembled at 23, 37 and 55 °C (*N* = 3, *n* = 3) with statistical significance indicating that present between CoAc _70_ and CoAc _30_ and other CoAcs. **** *p* < 0.0001; ns = not significant.

We combined pAmCyan (pDNA) and gelatin via electrostatic interaction in Milli-Q purified water to form a coacervate-based core. We varied the gelatin to a pDNA (pAmCyan) ratio first (Figure 2) and five gelatin to pDNA mass ratios were investigated (100:1, 70:1, 50:1, 30:1, 20:1 and 1:1 *w*/*w*). It was observed that a gelatin to pDNA mass ratio of 30:1 (CoAc_30_) or 70:1 (CoAc_70_) led to the formation of coacervates possessing significantly smaller diameters (170 nm and 151 nm, respectively) and smaller polydispersity indices (PDI) (0.17 and 0.21, respectively) compared to higher or lower ratios, which possessed particle diameters and PDIs of at least 257 nm and 0.32 in case of CoAc_100_ (Figure 2c,d). The intermediate-mass ratio of 50:1 (CoAc_50_) showed the largest particle diameter of 1772 and a PDI of 0.52 (Figure 2c,d). CoAc_30_ and CoAc_70_ showed slightly negative (−5.5 mV) and positive (1.8 mV) zeta potentials, respectively, while CoAc_50_ showed a zeta potential of almost zero (Figure 2b).

Under the coacervation conditions (MQ water, pH = 6.2, 37 °C) used for gelatin type A (Bloom number 228) and pDNA (4.7 kbp), the gelatin to pDNA mass ratios of 30:1, 50:1, and 70:1 provided the three coacervates with the smallest zeta potentials. Above 70:1 and below 30:1, a surplus of the positively charged gelatin or negatively charged pDNA existed in the coacervates. The repulsive forces between molecules of the similarly charged predominant polyion reduced the packing density of coacervate and its storage modulus, observed as an increase in particle size and PDI, in accordance with what was previously described by Arfin et al. [43]. A slightly overcharged coacervate, as in the case of CoAc_30_ and CoAc_70_, despite not having the highest packing density, or storage modulus is more kinetically stable than the high-density coacervates formed at the point of absolute charge neutralization (CoAc_50_), which are more liable to aggregation over time due to their surface neutrality [44]. This explains why CoAc_30_ and CoAc _70_ showed acceptable particle sizes and PDIs as opposed to CoAc_50_. It is worth mentioning that upon changing the plasmid size between 2.6 to 7.2 Kbp, such CoAc could still be assembled at an acceptable size and PDI at a mass ratio of 30:1 gelatin to pDNA (Figure S1).

Next, we optimized the core assembly temperature, where CoAc_100_ showed a smaller diameter of 190 nm and PDI of 0.26 when assembled at 37 °C compared to coacervates assembled at 55 °C or 23 °C showing diameters of 541 nm and 246 nm, and PDI of 0.611 and 0.35, respectively (Figure 2c,d). We found that using a gelatin to pDNA mass ratio of 30:1 and mixing temperature of 37 °C resulted in the smallest particle size and PDI, with a negative zeta potential. Hence, CoAc_30_ assembled at 37 °C was adopted for all subsequent experiments.

Our data suggest that the temperature of the solution during the initial interaction between pDNA and gelatin crucially affects core size and PDI [45]. Gelatin and pDNA both possess a helical conformation in aqueous media below 40 °C, with persistence lengths of 10 nm and 50 nm, respectively [46,47]. During the complexation of two polyions, the higher the chain flexibility of the two polyions, the better the interaction. The flexibility of a chain is an inverse function of its persistence length [47]. The persistence length of DNA was reported to be temperature-dependent, with a reduction in persistence length as the temperature increases [48]. Therefore, we assume that at 37 °C, pDNA has a smaller persistence length than it does at 23 °C, thus the proximity between the persistence lengths of gelatin and pDNA chains was higher at 37 °C than that at 23 °C, resulting in a better gelatin–pDNA interaction at 37 °C. The particles assembled at 37 °C had a smaller size and PDI compared to those formed at 23 °C. A mixing temperature of 55 °C produced CoAc_100_ with a significantly higher PDI and much larger particle size than the two other mixing temperatures. When the mixture was warmed to 55 °C, above the helix-coil transition temperature of gelatin [49], gelatin lost its helical structure, displaying a random coil conformation. We speculate that the loss of helical conformation compromised the structural synonymity of gelatin and pDNA, and despite pDNA possessing an even smaller persistence length and higher chain flexibility at this temperature, the coacervates formed between the pDNA’s helix and gelatin’s random coil were not as compact as the coacervates formed between pDNA’s and gelatin’s helices at 37 °C. Thus, the significance of maintaining the α-helical structure of gelatin type A by keeping the temperature of the mixture below the helix-coil transition threshold is critical for a successful initial interaction. This feature may also positively contribute to the stability of the system under physiological conditions.

#### 3.1.2. Core Stabilization

**Figure 3:**
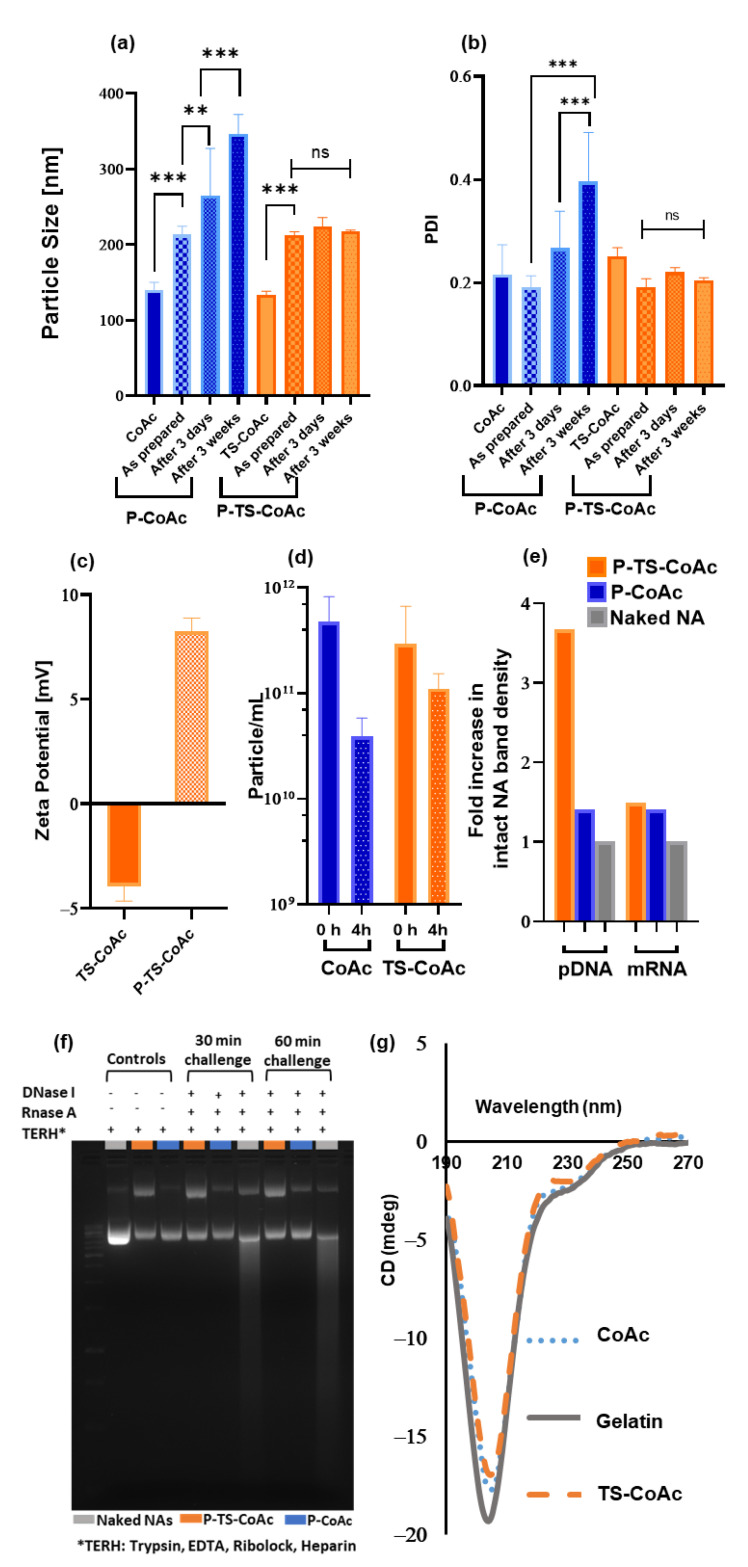
Assessment of the impact of thermal stabilization on particle stability after protamine sulfate coating with gelatin to protamine sulfate ratio of 5:1 (*w*/*w*). (**a**) Particle size (hydrodynamic diameter, nm) and (**b**) PDI of CoAc and TS-CoAc before and immediately after protamine sulfate coating, after 3 days and 3 weeks of storage, assessed using dynamic light scattering (DLS) (*N* = 3, *n* = 3). (**c**) Zeta potential of CoAc and TS-CoAc before and after protamine sulfate coating (*N* = 3, *n* = 3). (**d**) Colloidal stability of CoAc and TS-CoAc assessed using nanoparticle tracking analysis (NTA) as a function of particles mL^−1^ remaining after 4 h incubation in RPMI-1640 at 37 °C (*N* = 1, *n* = 3). (**e**) Densitometric analysis and (**f**) Gel migration assay following agarose gel electrophoresis of mCherry and pAmCyan either naked, loaded on P-TS-CoAc or P-CoAc, following either a 30 or 60 min incubation with DNase I/RNase A cocktail, the 60 min incubation point was used to generate figure (**e**). (**g**) Circular dichroism scans of gelatin, CoAc, and TS-CoAc in MQ water at 37 °C. ** *p* < 0.01, *** *p* < 0.001, ns = not significant.

Thermal treatment of CoAc_30_ via four subsequent heating–cooling cycles to form TS-CoAc led to a significant enhancement in the system’s colloidal stability. P-CoAc showed a progressive increase in particle diameter and PDI in MQ water starting as early as 3 days post coating, compared to the P-TS-CoAc, which resisted any core disruption by the protamine sulfate coat displaying no significant changes in particle diameter or PDI for 3 weeks (Figure 3a,b). The measured lower stability of CoAc-NPs when getting in contact with a competing polycation, such as protamine, could be expected in a core that solely relies on electrostatic interactions. Without further stabilization, protamine can easily displace gelatin from the core, which would typically result in an increase in particle size and PDI due to the release of heterogenous gelatin components from the entire nano-system, and hence the observed particle disruption in the case of P-CoAc and the lack of it in the case of P-TS-CoAc.

The successful deposition of protamine sulphate on the surface of TS-CoAc was accompanied by a reversal in zeta-potential from −3.9 mV in the case of TS-CoAc to 8.2 mV in the case of P-TS-CoAc (Figure 3c). The enhanced colloidal stability of TS-CoAc compared to CoAc was further confirmed as a function of particle count (particle mL^−1^) using NTA (Figure 3d and Figure S2). Following a 4h incubation of TS-CoAc in RPMI-1640, the particle count dropped from 2.9 × 10^11^ particles mL^−1^ to 1.1 × 10^11^ particles mL^−1^, on the other hand, CoAc decreased by one order of magnitude in particle count from 4.76 × 10^11^ particles mL^−1^ to 3.93 × 10^10^ particles mL^−1^.

Gel migration assay following DNase I/RNase A digestion of both P-TS-CoAc and P-CoAc coacervates demonstrated a superior protective effect of the thermally stabilized system (P-TS-CoAc) as opposed to the non-stabilized system (P-CoAC) with regards to the core loaded pDNA Figure 3e,f. Meanwhile, no detectable difference was observed between both the stabilized and unstabilized systems with regards to the surface-loaded mRNA’s shielding. Yet both nanocarriers showed superior protection of both the NA cargos compared to the naked NA control. This experiment could also provide insight into the release inducing mechanisms for the two NA cargos from the proposed system. As a combination of proteolytic activity and polyanion exchange is required for the release of these cargos and hence the use of Trypsin and Heparin as exemplary release inducers in this assay.

A trend in enhanced protection of the pDNA against serum nucleases following thermal stabilization was observed. In 10% serum incubation for 3 h, TS-CoAc maintained 31.9% of intact pDNA, compared to 17.4% of CoAc particles (Figure S5a,b).

Circular dichroism data (Figure 3g) showed a reduction in the negative ellipticity of the peak at 204 nm, reported to coincide with the random coil structure of gelatin [50]. The 204 nm peak intensity followed the rank order gelatin > CoAc > TS-CoAc when all were measured at 37 °C, indicating that coacervate formation and thermal stabilization caused a slight decrease in sample randomicity and promoted a more ordered structure. All samples were prepared from the same gelatin stock to eliminate any variations that may arise due to differences in concentration rather than optical activity among the samples.

Gelatin type A is the acidic denaturation product of collagen. Being a denatured protein, gelatin possesses low antigenicity [51], thus rendering it suitable for repeated administration. Gelatin possesses a lower charge density than most cationic polymers typically used in transfection, giving it a safety advantage [52], yet this renders its coacervates with nucleic acids much less stable. To date, techniques utilizing gelatin nanocarriers for NA delivery rely heavily on chemical cross-linking, even of cationized gelatin, for particle preparation and stabilization. Some of the commonly reported cross-linkers include symmetrical bifunctional aldehydes, such as glutaraldehyde and glyoxal [53], as well as EDC (1-ethyl-3-(3-dimethyl-aminopropyl) carbodiimide hydrochloride) [54]. In the specific scenario of nucleic acid delivery, such reactions may hinder the eventual release of the nucleic acid cargo from the gelatin matrix given the fact that both the nucleic acid and gelatin contain abundant amine groups and can thus become covalently bound. Covalently bound DNA-proteins are reported to hurdle the fidelity of gene expression in their host cell by interacting with the translation and transcription mechanisms of the cell via their DNA domain [55,56]. Thus, when designing our system, we aimed to avoid chemical cross-linking and to rely on the intrinsic properties of both gelatin and DNA to find an alternative stabilization technique. Gelatin–DNA coacervates rely mainly on easily reversible electrostatic interactions, hydrogen bonds, and hydrophobic interactions between gelatin and DNA. Such non-covalent interactions are suitable for the physiological interactions between nucleic acids and proteins during various cellular processes [57]. Gelatin–DNA coacervates have demonstrated an ability to irreversibly transform from a coacervate to an anisotropic nanogel phase upon heating above gelatin’s helix-coil transition temperature, followed by cooling below this temperature [58,59].

Thermal stabilization relies on the conversion of gelatin–pDNA coacervate in the case of CoAc to an anisotropic nanogel state (TS-CoAc), a phenomenon previously reported by Rawat et al. [58], but remains unused as a tool for nucleic acid delivery to date. When heating the gelatin above its helix-coil transition temperature of 40 °C, it assumes a predominantly random coil chain morphology, which extends further across the coacervate’s matrix, traversing more individual pDNA molecules along its length than a helix of equal length would. Upon sudden cooling of such coils in their extended state, the rapid loss of heat from the system is consumed in the formation of ionic and hydrogen bonds between the gelatin coils and pDNA, rather than allowing the coil a chance to resort back to its helix morphology. In this case, the pDNA acts as a scaffold supporting a matrix of extended gelatin coils even at a temperature below the helix-coil transition temperature. This physical method of cross-linking can be considered a safer option than most chemical cross-linkers commonly used for gelatin nanocarrier preparation, which compromise the intrinsic biodegradability and biocompatibility of the polymer [60,61]. Thus, we selected thermal treatment of gelatin–pDNA coacervates as our physical stabilization technique of choice.

#### 3.1.3. Shell Deposition

**Figure 4:**
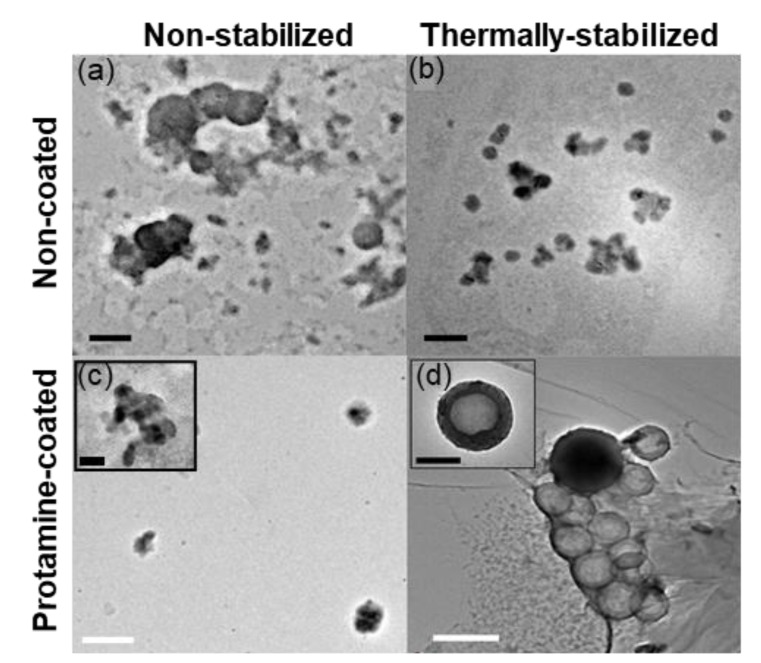
Transmission electron microscopy of unstained (**a**) CoAc (**b**) TS-CoAc (**c**) P-CoAc, (**d**) P-TS-CoAc, black bar = 200 nm, white bar = 500 nm.

A microfluidic system was adopted for coating the cores under laminar flow conditions. This technique deposited a homogenous coat across the whole particle population without any visible compromise to core integrity, as observed in the TEM images (Figure 4).

We could not detect a distinct core-shell structure in the case of P-CoAc (Figure 4c). Meanwhile, (Figure 4d and Figure S3) show the successful coating of the TS-CoAc with protamine sulfate, in protamine sulfate to gelatin mass ratio of 1:5, where an evident core-shell structure can be observed. This observation was further confirmed by the change in zeta potential from −3.9 mV to 8.2 mV (Figure 3c). The fact that P-TS-CoAc shows a distinct core-shell structure as opposed to the P-CoAc, further demonstrates the value of thermal stabilization.

Following mRNA surface-loading on P-TS-CoAc, particle size and PDI showed no discernible difference from unloaded P-TS-CoAc for up to one week. This further supports the assumption that the system remains intact and is taken up as a unit by the cells, while no mRNA-protamine sulfate coacervate sub-populations are formed (Figure S4).

Protamine is another peptide commonly used in the field of vaccination, that has been widely employed by CureVac AG in their RNactive^®^ technology, where it shows promise as an NA vaccine delivery tool [62,63,64,65,66]. Protamine is a naturally occurring membrane translocating peptide, with membrane translocation properties comparable to HIV-1 tat [67]. Protamine–DNA complexes bind to importins, which are transport proteins associated with the nuclear pore complex, thus facilitating the nuclear translocation of pDNA [38,68]. All the aforementioned properties in addition to its established pharmaceutical application became our motivation to use it as a particle coating.

### 3.2. Entrapment Efficiency and Nanocarrier NA Capacity

Entrapment efficiency (EE%) assessed using PicoGreen assay (Table 2) showed that pDNA was fully incorporated into the system at a gelatin to pDNA mass ratio as low as 30:1. Both CoAc_30_ and TS-CoAc_30_ showed no considerable difference in EE % of pDNA (Table 2). RiboGreen assay performed on P-TS-CoAc showed EE% of 97.81% of mRNA. The carrier packed more mRNA (1.884 × 10^12^) than pDNA (1.076 × 10^12^) molecules per 170 µg of particles. Moreover, on the particle level, based on the average particle count obtained using NTA (data not shown) each P-TS-CoAc particle packed approximately 5318 pDNA molecules and 9312 mRNA molecules. Calibration curves used in the establishment of these assays are provided in (Figure S6).

### 3.3. Cytotoxicity Assay

**Figure 5:**
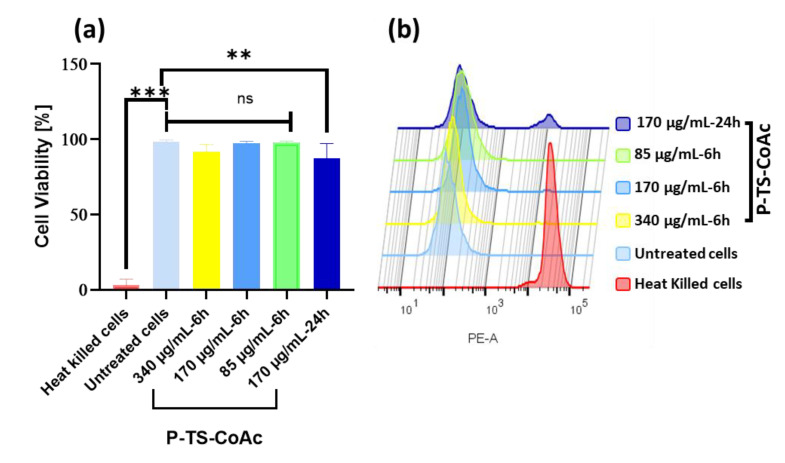
Cytotoxicity assay of P-TS-CoAc in DC2.4 murine dendritic cell line using fixable dead stain (568/583) (**a**) % Cell viability following 6 h incubation of P-TS-CoAc (340,170 or 85 µg/mL) or 24 h incubation of P-TS-CoAc (170 µg/mL) (*N* = 3, *n* = 3); ** *p* < 0.01, *** *p* < 0.001, ns = not significant. (**b**) Fluorescence intensity (dead stain uptake) of cells following different treatments.

Compared to untreated cells, murine dendritic cell line (DC2.4) cells treated with either the two-fold (340 µg·mL^−1^), the same (170 µg·mL^−1^) or half of (85 µg·mL^−1^) the particle concentration used in transfection efficiency studies showed cell viabilities of 91.9%, 97.1%, and 97.7%, respectively, following 6 h incubation (Figure 5). Meanwhile, a 24 h incubation of 170 µg/mL particles showed 87.4% cell viability. A total of 170 µg/mL was selected for the 24 h extended viability assay because it was the dose to be used for the transfection trials. Protamine–mRNA–pDNA coacervates assembled using either equivalent protamine doses in P-TS-CoAc concentrations of 340, 170 or 85 µg/mL or 5-fold; these concentrations were also investigated and showed no significant difference in cytotoxicity compared to either P-TS-CoAc or untreated controls following 6 h incubation (Figure S7). These data align with the established biocompatibility of the two major nanocarrier components, gelatin and protamine sulfate [60,61].

### 3.4. Transfection Efficiency of Co-Delivered mRNA (mCherry) and pDNA (pAmCyan1) in Murine Dendritic Cell Line DC2.4

**Figure 6:**
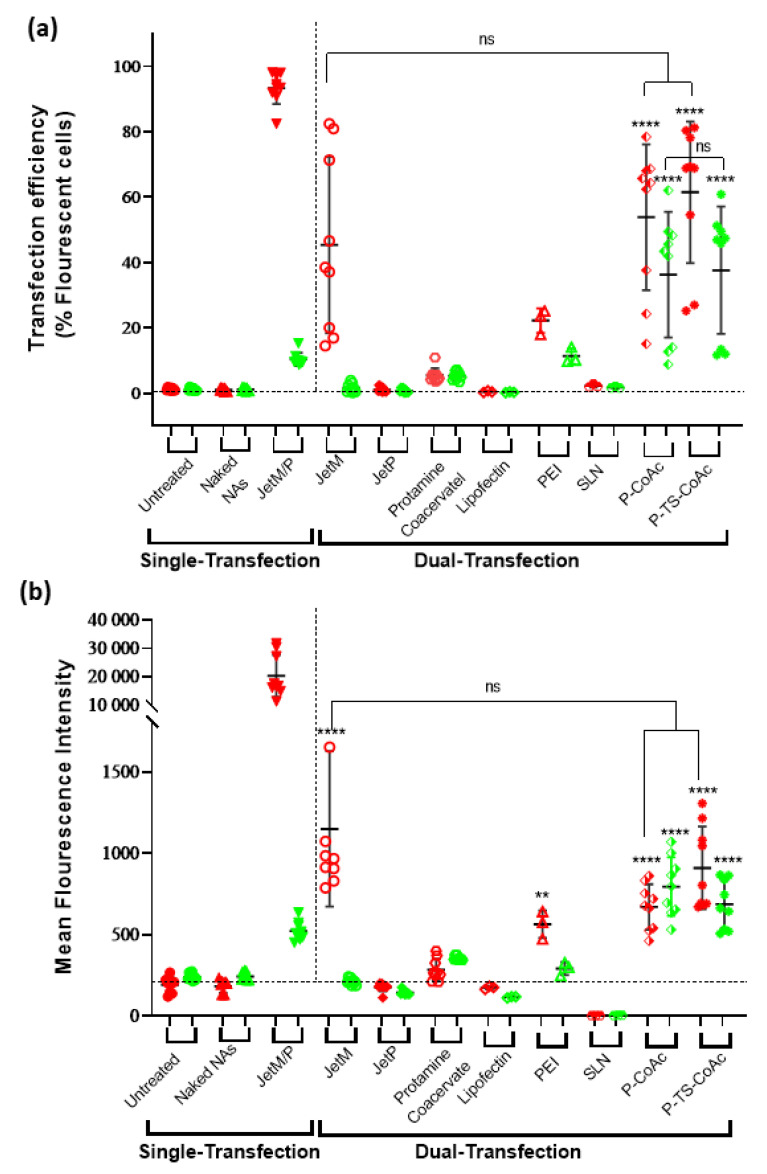
Flow cytometric assessment of (**a**) transfection efficiency and (**b**) mean fluorescence intensity (MFI) of pAmCyan and mCherry loaded on P-CoAc and P-TS-CoAc, compared to single transfection using either JetMessenger for mRNA or JetPrime for pDNA or double transfection using both mRNA and pDNA with either JetMessenger, JetPrime, protamine sulfate (*N* = 3, *n* = 3), Lipofectin, PEI or SLN (*N* = 1, *n* = 3) coacervate in murine dendritic cell line DC2.4; ** *p* < 0.01, **** *p* < 0.0001, ns = not significant.

Upon application of both protamine sulfate coated and mRNA surface-loaded P-CoAc and P-TS-CoAc to murine dendritic cell line DC2.4, both P-CoAc and P-TS-CoAc showed successful, simultaneous transfection of the cells with both mRNA (mCherry) and pDNA (pAmCyan). The transfection efficiency and level of protein expression of both pAmCyan and mCherry significantly surpassed all other test groups except for the JetM single transfection of mCherry transfection. Yet in the case of double transfection, despite the insignificant difference in transfection efficiency between JetM and both P-TS-CoAc and P-CoAc, JetM failed to cause any discernible transfection with pAmCyan (Figure 6a,b). Figure 6a,b show comparable transfection efficiencies and AmCyan expression levels in the case of pDNA, with either P-TS-CoAc and P-CoAc. P-TS-CoAc showed a transfection efficiency of 37.6 ± 19.45% and an MFI of 686 ± 148 as opposed to 36.22 ± 19.21% and 670 ± 139 with P-CoAc, thus indicating that thermal stabilization did not reduce the transfection efficiency of pDNA. Both systems were more efficient than the commercial transfection reagent JetPrime (0.64 ± 0.57%, 152 ± 43), given the challenging nature of transfection in DC2.4. P-TS-CoAc showed a transfection efficiency and MFIs of 61.4 ± 21.6% and 909 ± 253 as opposed to 53.8 ± 22.3% and 794 ± 180 with P-CoAc for mRNA. We could resort the difference, though statistically insignificant, to the more efficient coating and more stable surface in the case of P-TS-CoAc, which allowed better binding and stabilization of the surface-loaded mRNA.

Protamine sulphate-NA coacervate serving as an internal control showed inconsistent transfection of both pDNA and mRNA (Figure 6), which was negligible in most samples. JetMessenger (for mRNA) and JetPrime (for pDNA) were used as representatives of successful, widely applied commercial transfection reagents, which could serve as positive controls. Yet when used for co-transfection with both mRNA and pDNA, both systems displayed negligible transfection for the NA they were not designed to deliver, as well as a reduction in the transfection efficiency of their NA of specialization as opposed to our systems that demonstrated successful co-transfection. The same holds for Lipofectin, PEI, and DLin-MC3-DMA based SLN (Figure 6a).

Protamine combines an ability to promote cytosolic delivery of mRNA, as well as nuclear translocation of pDNA via four specialized nuclear localization signal-like sequences in its structure [69,70], setting it apart from most of the aforementioned controls. However, when a protamine coacervate was assembled at the same protamine:mRNA:pDNA mass ratios as P-TS-CoAc and used as an internal control using the same NA doses, the transfection performance of the protamine coacervate was inferior to P-TS-CoAC. This could be resorted to a trojan horse effect exerted by gelatin–pDNA coacervate core, because, in such an arrangement, a considerable fraction of the anionic charges of pDNA could be occluded within the gel core, while only a fraction of the surface-exposed pDNA interacts with the protamine coat, sparing more of protamine’s cationic groups for endosomal disruption. Such an arrangement could be allowing the protamine to function at an apparently higher NP ratio despite the lower actual protamine dose. An additional possible explanation for the superior dual transfection performance of P-CoAc and P-TS-CoAc compared to the controls may also be due to a unique time-resolved release and translation of NA cargo from these two systems; this could hence be the subject of a more detailed future study.

**Figure 7:**
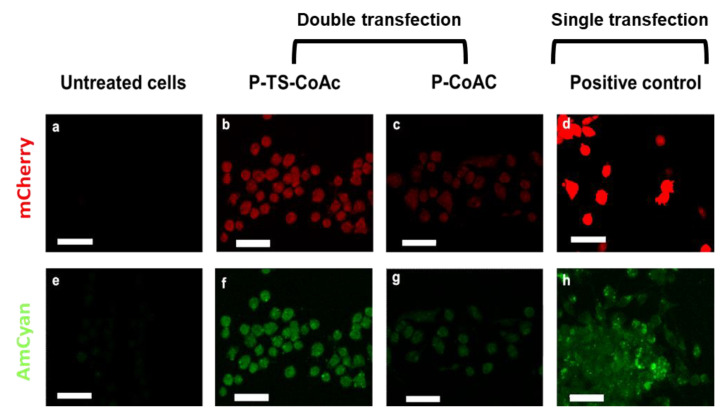
Assessment of transfection efficiency and gene expression of mCherry (red fluorescent reporter) and pAmCyan (green fluorescent reporter) in DC2.4 after 6 h of treatment of samples or controls followed by 48 h incubation (**a**) confocal laser scan microscopy showing expression of mCherry (red) and AmCyan(green) in DC2.4 cells treated with (**b**,**f**) P-TS-CoAc, (**c**,**g**) P-CoAc, (**d**) JetMessenger, (**h**) JetPrime compared to (**a**,**e**) untreated cells; the white bar = 39.64 µm. CLSM images are shown with 40% increased brightness from the original images (obtained with identical laser intensity settings for all samples).

Moreover, the confocal microscopy images of the highest performing treatments reveal that cells treated with P-TS-CoAc displayed visibly more consistent transfection patterns in the case of mRNA (Figure 7b) and pDNA (Figure 7e) than cells treated with the P-CoAc (Figure 7c,g). This might indicate that the enhancement in colloidal stability of the system via thermal stabilization, in addition to providing better shielding of the core-loaded pDNA and a more stable surface for a stable loading of mRNA, allowed the system to remain intact for longer during transfection and for the co-loaded NAs to be taken up as a unit, a feature that can prove valuable in a vaccination context of antigen-adjuvant co-delivery. This observation suggests that thermal stabilization may have enhanced both the transfectional and co-transfectional capabilities of the system. Cells treated with commercial transfection reagent displayed a strong expression of mCherry in the case of JetMessenger (Figure 7d) and a weaker yet more homogenous expression of AmCyan in the case of JetPrime (Figure 7h). A detailed gating strategy is provided in (Figure S8).

## 4. Conclusions

This study shows an approach to produce nanocarriers based on gelatin, a pharmaceutically established biopolymer, using a mild and straightforward preparation technique to load polynucleic acid cargos suitable for gene delivery. The shown improvement of colloidal stability by thermal stabilization could be essential for the further development of a product with sufficient storage stability. As expected for a system made from a biocompatible, biodegradable protein material, no cytotoxicity was observed in the concentration range successful for transfection.

A core-shell system was prepared by taking advantage of an intrinsic property of the two core components, gelatin and pDNA, to form an irreversible gel when heated together. The stability of this gel-core allowed for the deposition of a protamine sulfate shell. We loaded mRNA on the particle’s shell while maintaining pDNA in the core. Transfection of both nucleic acids was observed with comparable transfection efficiencies from both pDNA and mRNA when used in a mass ratio of 5:1, as opposed to clinical, experimental, and commercial transfection reagents, where such co-transfection was not feasible.

We here present a proto-type NA carrier with unique co-transfectional capabilities. A vast pool of applications can be based on or expanded off the concept, both in the areas of vaccine delivery, as well as protein replacement therapies. In this study, we employed commercial fluorescent reporter molecules of rapid onset of expression and long-expression product half-life. Using selected combinations of nucleotides, we think the interesting possibility of gene expression at varying time scales could be achievable and should be further studied. In future studies, the potential of this system to optimize the expression kinetics and location of NA cargos could be explored. Besides clinical applications, this system could also serve as a research tool to study differences between expression kinetics of more than one NA cargo in parallel.

## Figures and Tables

**Table 1:** Composition and nomenclature of different formulations.

Sample	Gelatin Concentration [mg·mL^−1^]	DNA Concentration [µg·mL^−1^]	Gelatin to DNA Ratio [*w*/*w*]	Protamine Sulphate Concentration [mg·mL^−1^]	Protamine Sulphate to Gelatin Ratio [*w*/*w*]
CoAc_100_	10	100	100:1	–	–
CoAc_70_	7	100	70:1	–	–
CoAc_50_	5	100	50:1	–	–
CoAc_30_^1^	3	100	30:1	–	–
CoAc_20_	2	100	20:1	–	–
CoAc_1_	0.1	100	1:1	–	–
TS-CoAc ^2^	3	100	30:1	–	–
P-CoAc ^3^	3	100	30:1	0.3	1:5
P-TS-CoAc ^2,3^	3	100	30:1	0.3	1:5

^1^ CoAc_30_ was selected for further experiments and is referred to as CoAc without a subsequent numerical value in the lower part of the table and throughout the text; ^2^ TS-CoAc is CoAc_30_ subjected to four heating–cooling cycles; ^3^ “P-’’ indicates protamine sulfate coating.

**Table 2:** The entrapment efficiency (EE %) of the carriers for pDNA (pAmCyan) was assessed using PicoGreen assay, whereas RiboGreen assay was used for mRNA (mCherry) (*N* = 1, *n* = 3). The average number of pDNA or mRNA molecules per particle dose (170 µg as the dose used per well in a 24 well-plate format) and the numbers of pDNA or mRNA molecules per particle (NP) were calculated based on the used amount of NAs and the particle count from NTA.

	pAmCyan			mCherry		
Sample	EE [%]	Molecules/Dose	Molecules/NP	EE [%]	Molecules/Dose	Molecules/NP
CoAc	100.10 ± 0.28%	1.076 × 10^12^	5318	No colloidally stable coated P-CoAc for surface-loading		
(P-)TS-CoAc	100.12 ± 0.39%	1.076 × 10^12^	5318	97.81 ± 1.06	1.884 × 10^12^	9312
jetPrime ^a^	100.01 ± 9 × 10^−5^%	1.076 × 10^12^	-	-	-	-
JetMessenger ^b^	-	-	-	100.66 ± 20.94%	1.884 × 10^12^	-

^a,b^ For the transfection reagents JetPrime and JetMessenger, the particle count was not available to calculate the number of NA molecules/NP.

## Data Availability

Data is contained within the article or Supplementary Material.

## Supplementary Materials

The following are available online at https://www.mdpi.com/article/10.3390/pharmaceutics13111924/s1, Figure S1: DLS assessment of CoAc and TS-CoAc assembled using pDNA of varying sizes., Figure S2: Screenshots of NTA analysis of CoAc and TS-CoAc immediately after addition to RPMI-1640 at 37 °C in a ratio of 1:10 *v*/*v*, and after 4 h incubation in such medium., Figure S3: Transmission electron microscopy of unstained P-TS-CoAc, Figure S4: Particle sizes (nm) and PDI of P-TS-CoAc before loading with mCherry, 15 min after loading with mCherry and 7 days after loading with mCherry. Figure S5: Gel electrophoresis for assessment of serum stability of pAmCyan cargo in coacervate and TS-CoAc following 3 h incubation in 10% serum, Figure S6: PicoGreen and RiboGreen assays’ calibration curves, Figure S7: Cytotoxicity assay of protamine coacervate, Figure S8: Gating information of DC2.4 during the in-vitro transfection assay.
