# Supplementary material for: Co-Delivery of mRNA and pDNA Using Thermally Stabilized Coacervate-Based Core-Shell Nanosystems
Source: Pharmaceutics. 2021 Nov 13;13(11):1924. doi: 10.3390/pharmaceutics13111924 (PMC8619316; doi:10.3390/pharmaceutics13111924)
Supplement: Supplementary file 1 [file pharmaceutics-13-01924-s001.zip › pharmaceutics-1388193-supp-update.pdf]

pharmaceutics

Supplementary Materials: Co-delivery of mRNA and pDNA Using Thermally Stabilized Coacervate Based Core-Shell Nanosystems

Sarah S. Nasr, Sangeun Lee, Durairaj Thiyagarajan, Annette Boese, Brigitta Loretz and Claus-Michael Lehr

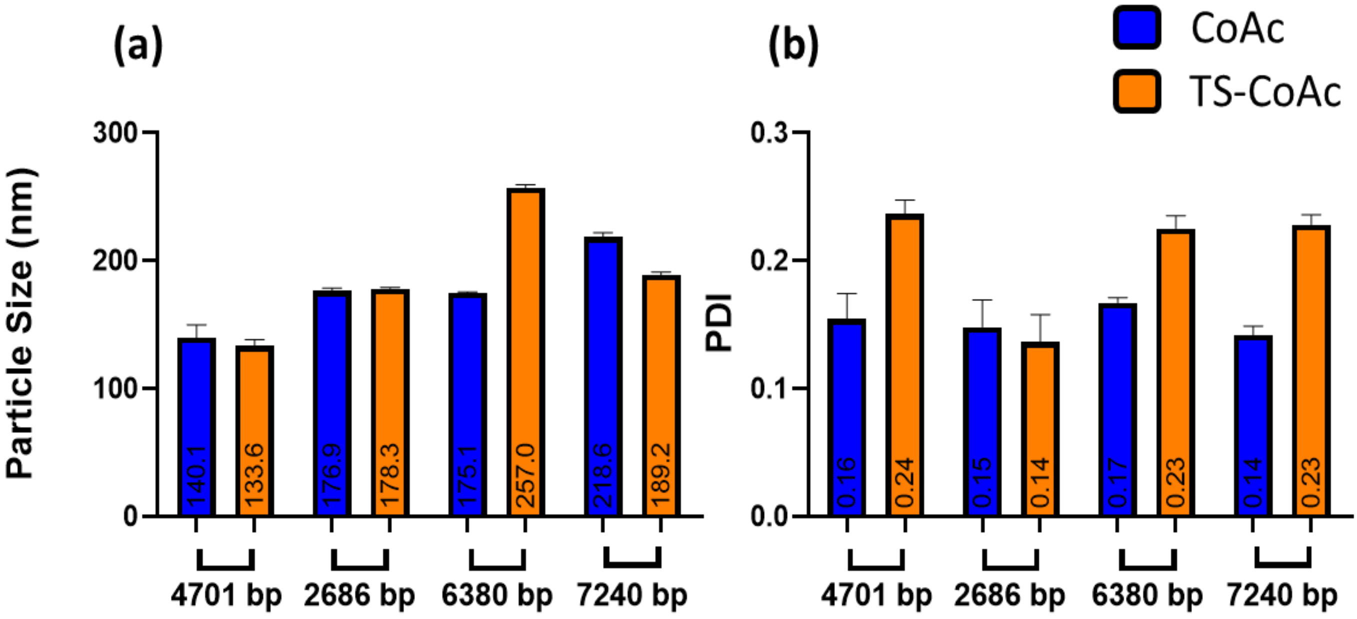

Figure S1. (a) Particle size and (**b**) PDI of CoAc and TS-CoAc assembled using pDNA of varying sizes at gelatin to pDNA mass ratio of 30:1 assessed using dynamic light scattering (DLS).

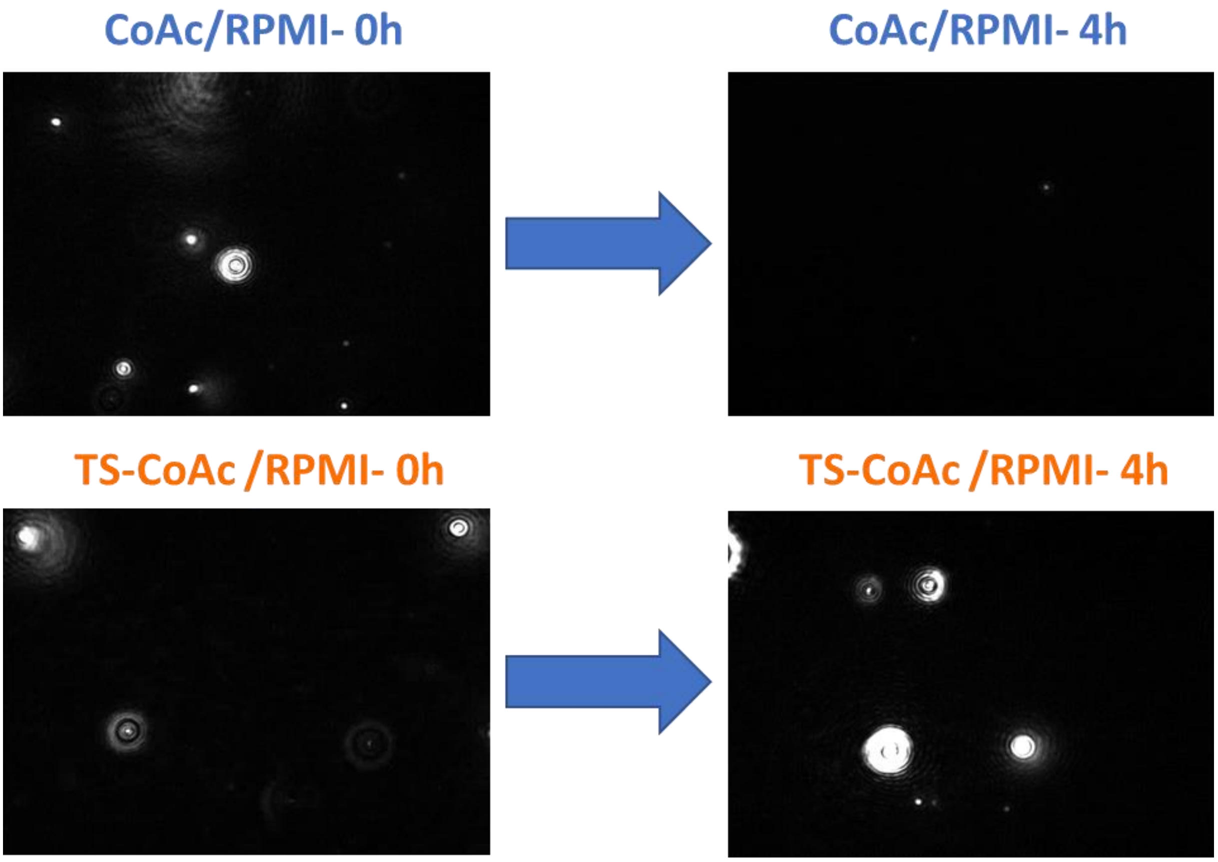

Figure S2. Screenshots of nanoparticle tracking analysis (NTA) of CoAc and TS-CoAc immediately after addition to RPMI-1640 at 37 °C in ratio of 1:10 v/v, and after 4 h incubation in the medium.

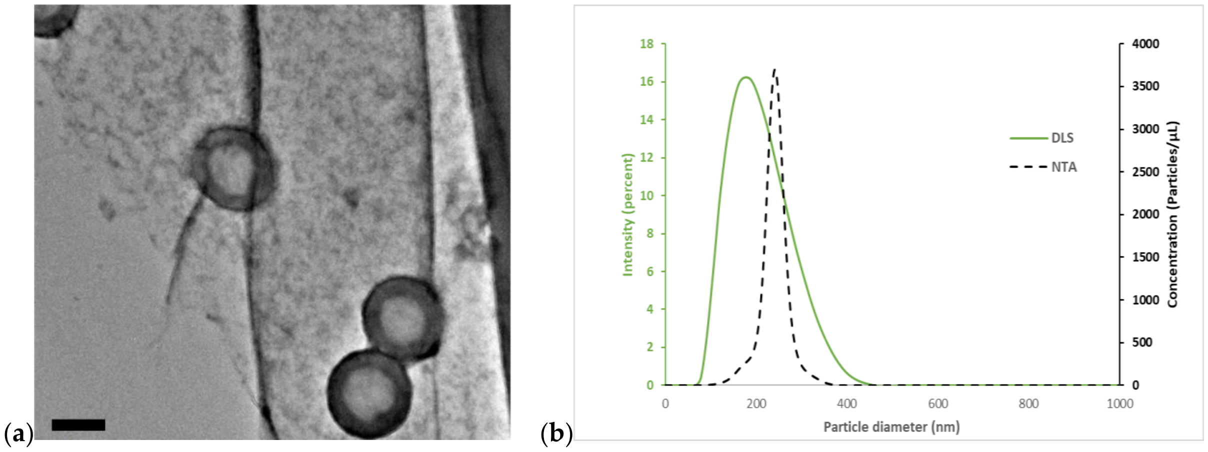

Figure S3. (**a**) Transmission electron microscopy (TEM) of unstained P-TS-CoAc, black bar = 200 nm. (**b**) Particle size and size distribution of P-TS-CoAc measured using DLS and NTA.

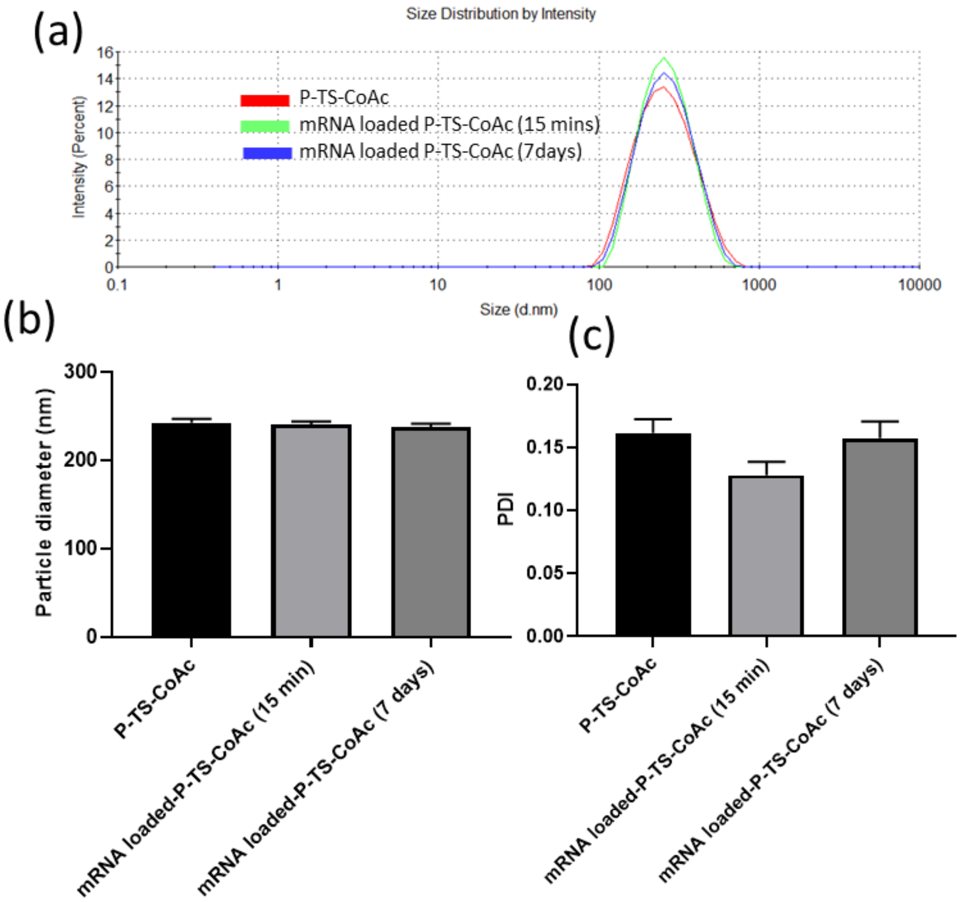

Figure S4. (**a**) Particle size distribution by intensity characterized using DLS before, 15 min after, and 7 days after the surface loading of P-TS-CoAc with mCherry. (**b**) Particle sizes (hydrodynamic diameter, nm) and (**c**) PDI of P-TS-CoAc before loading with mCherry, 15 minutes after loading with mCherry and 7 days after loading with mCherry.

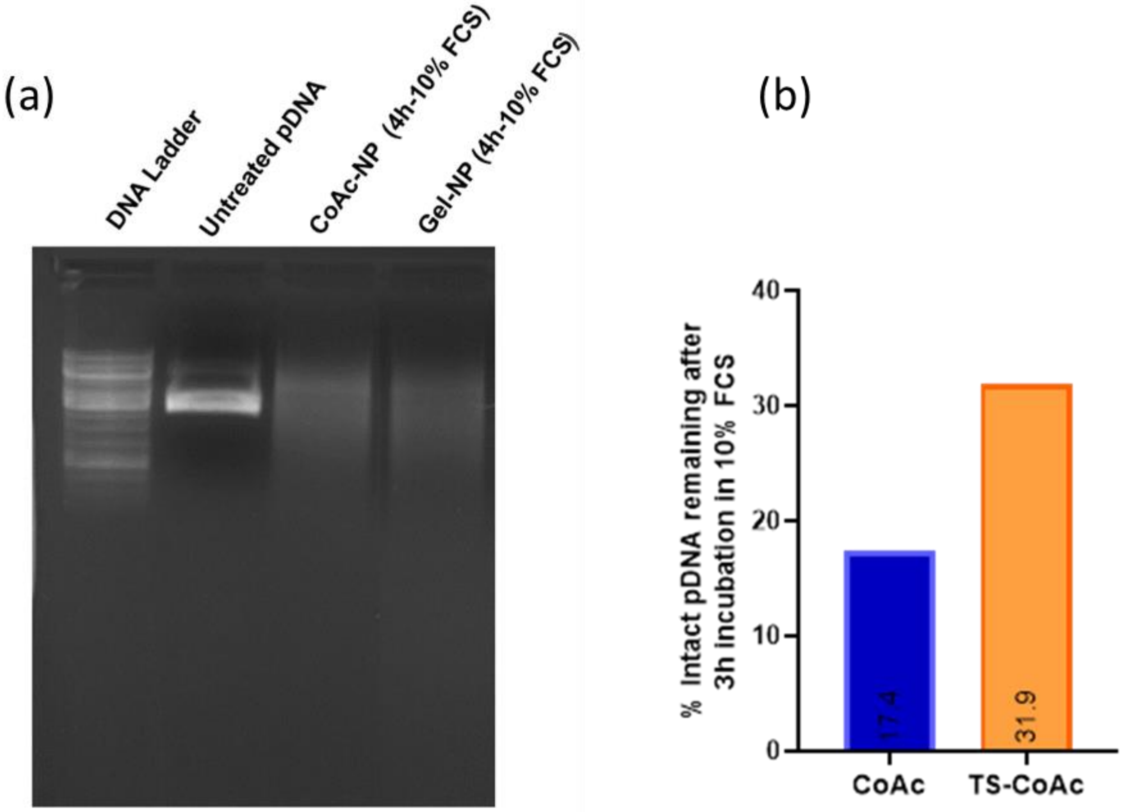

Figure S5. (**a**) Gel electrophoresis for assessment of serum stability of pAmCyan1 cargo in coaceravate and TS-CoAc following 3 h incubation in 10 % fatal calf serum (FCS). (**b**) Densitometric analysis of pDNA bands normalized to band intensity of the equivalent amount of pDNA in TBE buffer; the pDNA cargo was released from CoAc or TS-CoAc by trypsin and high molecular weight heparin, following 3 h incubation with 10% FCS in HBSS.

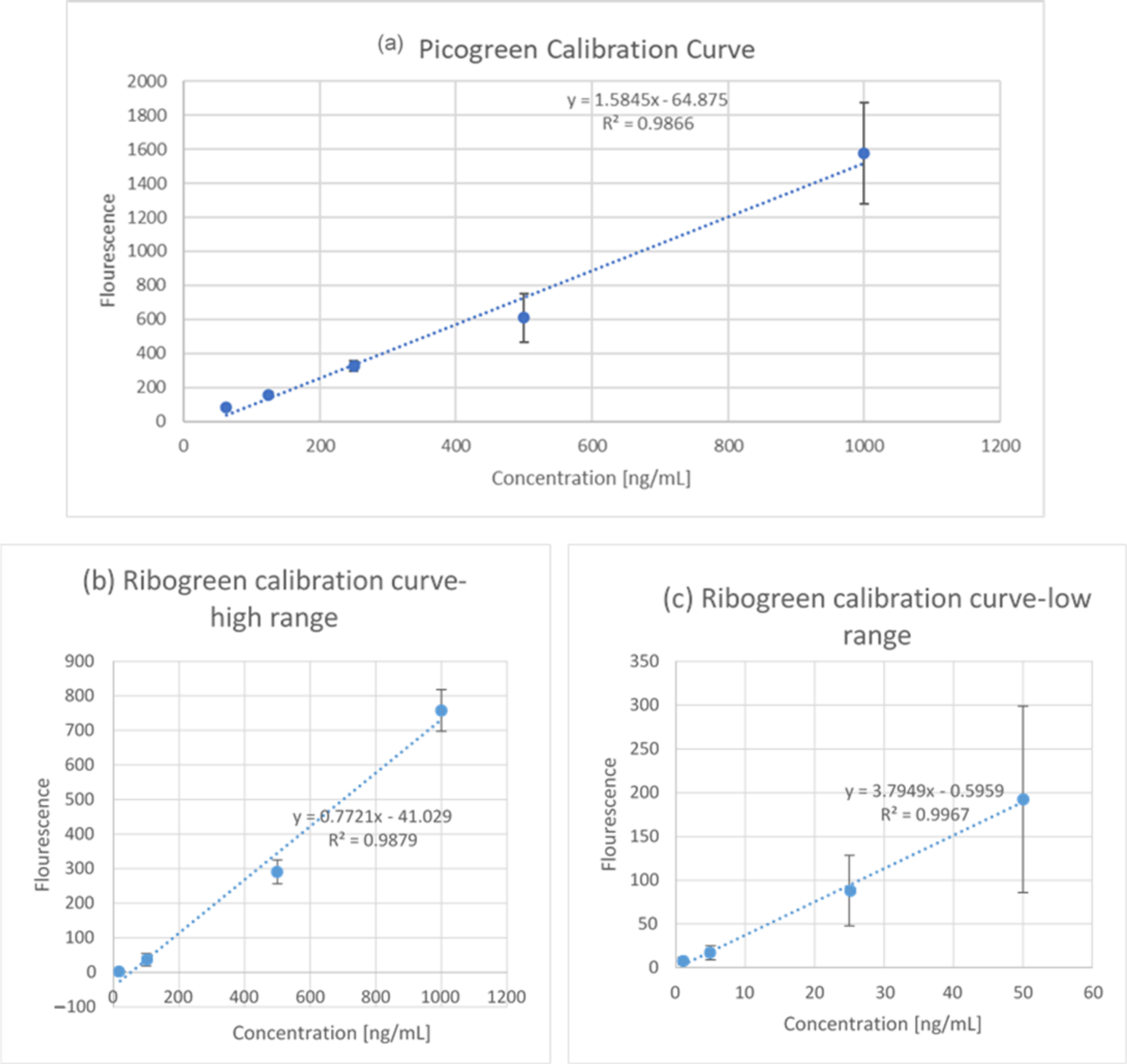

Figure S6. (**a**) Picogreen assay calibration curve, pAmCyan1 concentration range from 31.5–1000 ng/mL (N = 3,n = 1) , Ribogreen assay calibration curves (**b**) high range with mCherry concentration range from 20–1000 ng/mL (**c**) low range with mCherry concentration range from 1–50 ng/mL (N = 3,n = 1).

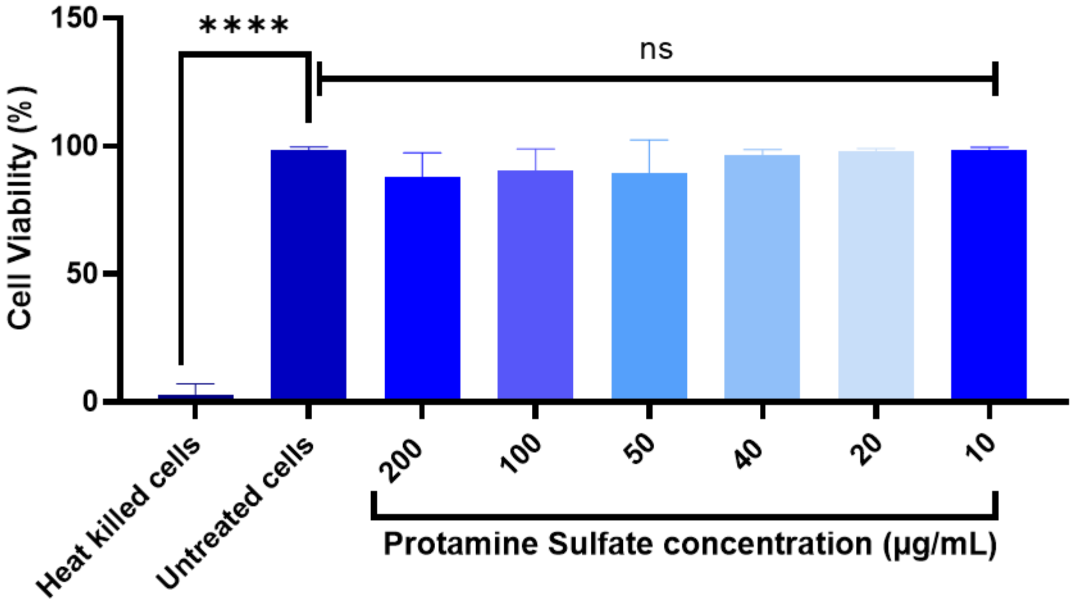

Figure S7. Cytotoxixity assay of protamine coacervate with mCherry and pAmCyan1 at equivalent doses to 0.5× (10 µg/mL), 1× (20 µg/mL), 2× (40 µg/mL), 2.5× (50 µg/mL), 5× (100 µg/mL) and 10× (200 µg/mL) P-TS-CoAc protamine coat mass using 170 µg/mL particle dose. **** $p < 0.0001$; ns = not significant.

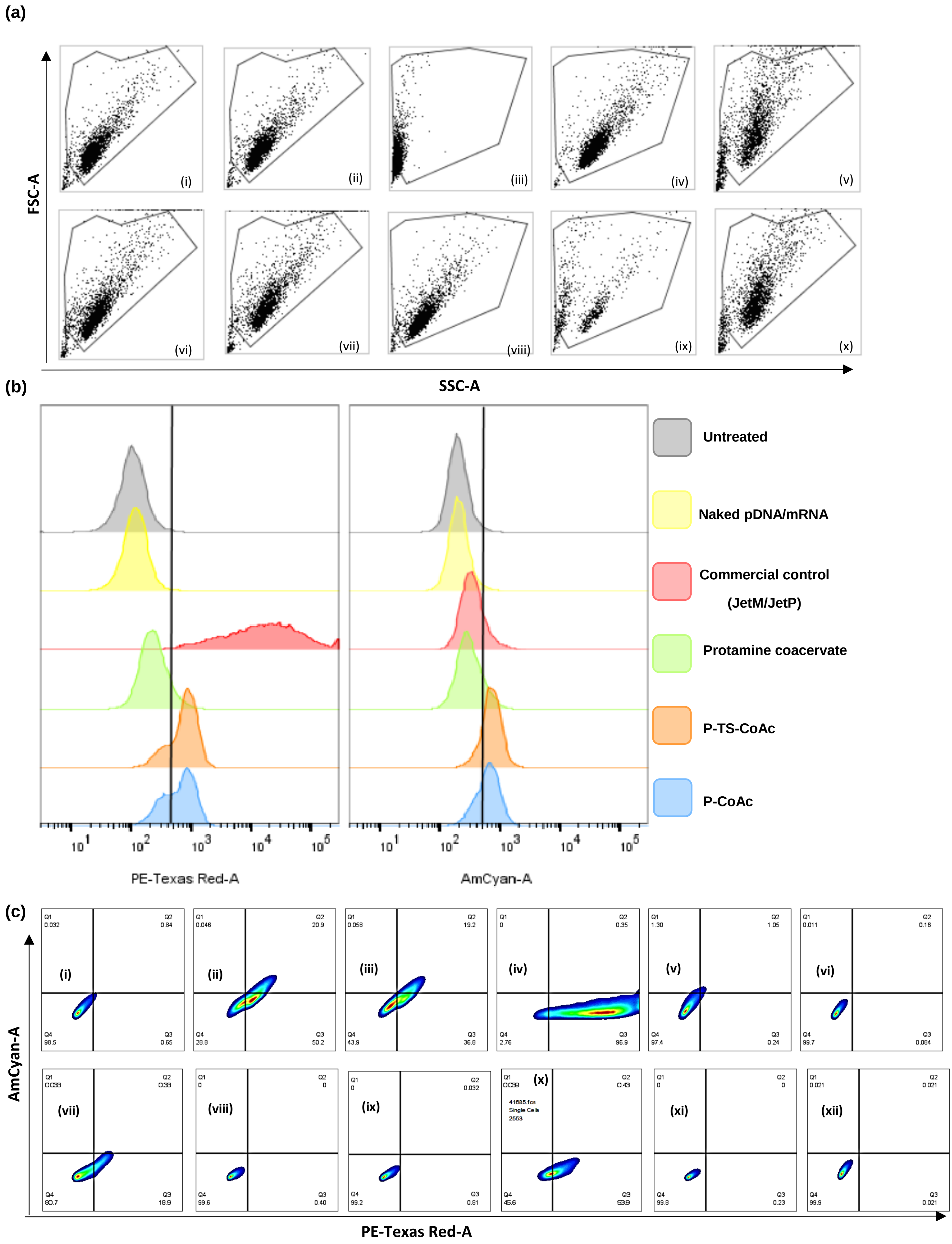
(a)
FSC-A
SSC-A
(i)
(ii)
(iii)
(iv)
(v)
(vi)
(vii)
(viii)
(ix)
(x)
(b)
PE-Texas Red-A
AmCyan-A
Untreated
Naked pDNA/mRNA
Commercial control
(JetM/JetP)
Protamine coacervate
P-TS-CoAc
P-CoAc
(c)
AmCyan-A
PE-Texas Red-A
(xi)
(xii)

Figure S8. (**a**) Dot plots and gating of DC2.4 with different treatments (i) untreated, (ii) protamine coacervate, (iii) JetM-single transfection, (iv) JetP-single transfection, (v) JetM-dual transection, (vi) JetP-dual transfection, (vii) Lipofectamine-dual transfection, (viii) PEI dual transfection, (ix) P- Co-Ac, and (x) P-TS-CoAc. (**b**) Histograms of DC2.4 with different traetements showing shift in flourscence intensity along the PE-Texas Red-A and AmCyan-A channel. (**c**) Quadrant gating of DC2.4 with different treatemnets (i) untreated cells, (ii) P-TS-CoAc, (iii) P-CoAc, (iv) JetM-single transfection, (v) JetP-single transfection, (vi) naked pAmCyan1 and mCherry, (vii) PEI-double transfection, (viii) Lipofectamine double transfection, (ix) protamine coacervate-double transfection, (x) JetM-double transfection, (xi) JetP-double transfection, and (xii) SLNs-double transfection.
